# Importance of transoesophageal echocardiography in preventing complications due to intraoperative dislodgement of left atrial thrombus

**DOI:** 10.4103/0019-5049.72655

**Published:** 2010

**Authors:** Rahul Guru

**Affiliations:** Department of Anaesthesia, Cork University Hospital, Wilton, Cork, Republic of Ireland, Ireland

Sir,

A 61-year-old man presented with central chest tightness. Although the Electrocardiogram (ECG) did not show ischemic changes, the troponin was raised. He was in fast atrial fibrillation with haemodynamic compromise. He was given two Direct Current (DC) shocks after which it converted to sinus rhythm. Cardiac risk factors included Diabetes Mellitus (DM), hypercholestrolaemia and smoking. A Transthoracic Echocardiography (TTE) and a Coronary Angiography was planned.

Transthoracic Echocardiography revealed the following findings — 1. Left Ventricle (LV) borderline dilated; 2. LV systolic function moderate to severely reduced; 3. Severe posterior wall hypokinesia; 4. Ejection Fraction (EF) of 25 to 35%; 5.Heavily calcified aortic valve with poor cusp excursion. Max systolic gradient=20 mmHg.

Cardiac Catheterisation confirmed these findings and showed significant three-vessel coronary artery disease.

It was planned to do a Coronary Artery Bypass Graft with / without Aortic Valve Replacement (AVR). Transoesophageal Echocardiography (TOE) was performed (four days after performing the TTE), on the evening prior to the surgery, to assess whether an AVR should be done. It was decided to do a coronary artery bypass graft (CABG) plus AVR. Incidentally a thrombus in the left atrial appendage was found [Figure 1].

**Figure 1:**
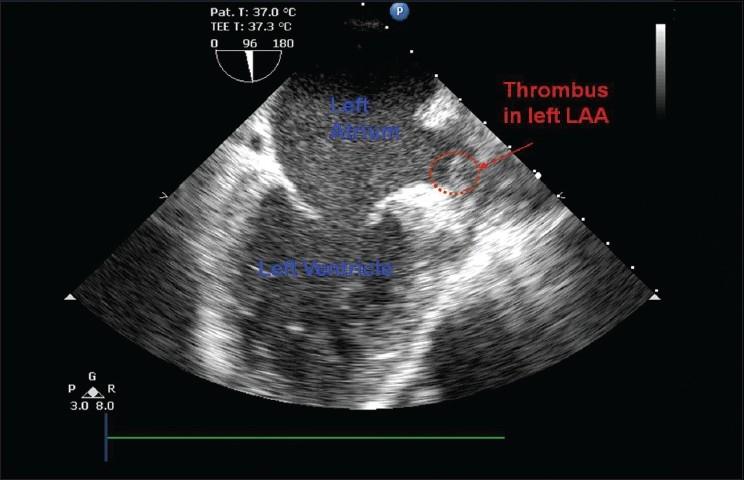
TOE image showing the left atrium and the left ventricle and the thrombus in the left atrial appendage

On the operation day the patient was taken to the theatre. Along with the usual monitoring for cardiac surgery a transoesophageal echo (TOE) probe was also inserted. Surgery started, sternotomy and pericardiectomy were done and the heart was exposed. Prior to handling of the heart, the presence of a thrombus in the Left Atrial Appendage (LAA) was confirmed by TOE. The surgeons aimed to minimise the manipulation of the heart to avoid the thrombus from dislodging. Aortic cannulation was done and prior to the right-sided cannulation the surgeons were considering bicaval cannulation rather than right atrial cannulation, in an attempt to minimise the manipulation of the heart. During this time they enquired regarding the thrombus. The thrombus was visible in the Left Atrial Appendage at this time. Suddenly the thrombus dislodged from the appendage and disappeared from our view on the echocardiogram [Figure 2]. We then saw it tumbling in the left ventricle for a few seconds [Figure 3] after which it disappeared into the circulation. The surgery was continued.

**Figure 2:**
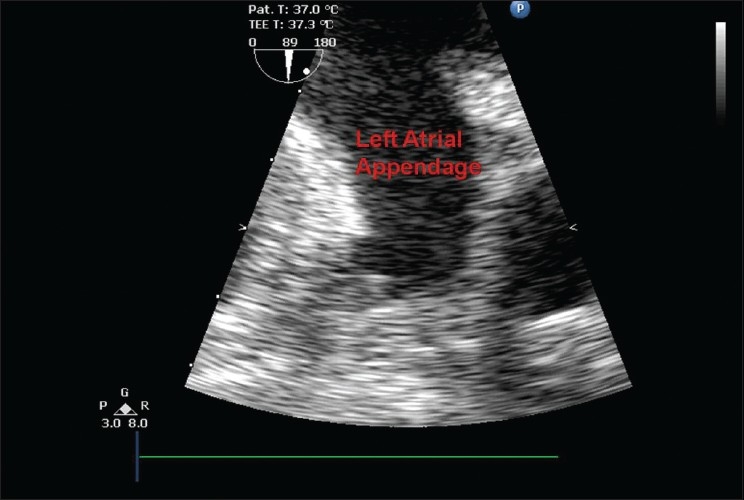
TOE image showing an empty left atrial appendage after the thrombus had migrated

**Figure 3:**
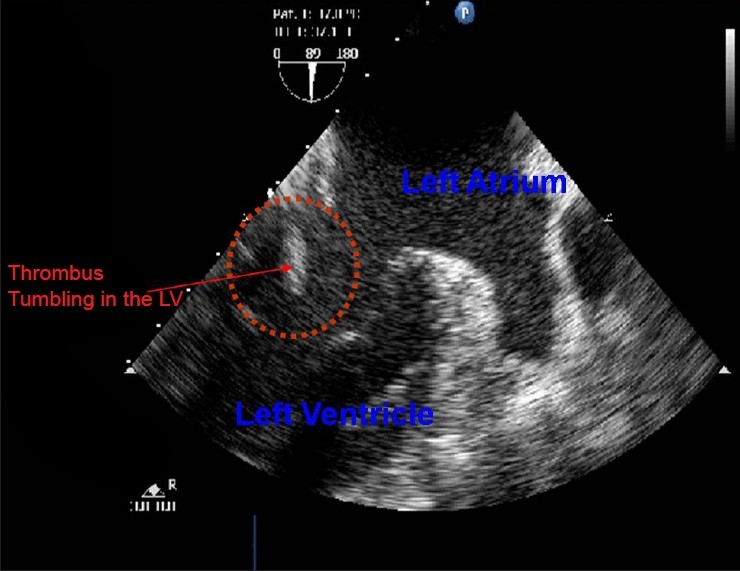
TOE image of the left atrium and ventricle with the thrombus seen tumbling in the left ventricle. It was seen in the ventricle for a few seconds before it disappeared

As the thrombus had dislodged into the circulation a search was made for it after the CABG plus AVR had been done. A carotid ultrasound and examination of peripheral pulses was performed. The left leg below knee was found to be cold and the pulse was less. Vascular surgeons were called to review and they decided to do a Popliteal Embolectomy. The thrombus was found in the Popliteal Artery and was removed. This was followed by a return of good pulse in Popliteal A and Anterior Tibial A.

Postoperatively the patient was transferred to the intensive care unit (ICU), weaned and woken up. He was alert and oriented. There was no focal neurological deficit. Haemodynamically he was stable with peripheral pulses present. The left foot was warm and well perfused. On post operative day one he was transferred to the ward. A few days later he was discharged home.

There are no guidelines for the management of a left atrial thrombus seen pre-operatively.

Theoretically there are a few management options

1. Heparin
2. Thrombolysis — Carries a high risk of systemic embolism
3. Surgical removal

The patient recently had a myocardial infarction (MI) and also had an aortic stenosis. Giving heparin the night before surgery is not a feasible option. Although postponing surgery would have carried a high risk to the patient.

On the other hand thrombolysis causes the thrombus to lyse into small particles that can embolise into the system causing a stroke and organ and limb ischaemia. As the patient was going for surgery the very next day it was decided to proceed without any change in the plan.

During the surgery the surgeons would try to minimise the manipulation of the heart with the anaesthetist keeping an eye on the thrombus with the help of TOE. Unfortunately the thrombus did embolise, but only to the leg.

As we were able to visualise this on TOE we started looking for signs of embolisation as soon as the surgery was over. A carotid ultrasound and examination of the peripheral pulses were done in the diagnostic workup.

Echocardiography has proven to be a useful tool in the diagnosis and evaluation of cardiac masses. TOE, when used during cardiac surgery, has been shown to influence surgical and medical management.[1–3]

In this case TOE helped us to actually see the thrombus migrating. This made us look for the signs of embolism and manage them early.

## References

[1] Kneeshaw JD (2006). Peri-operative TOE- does it have an effect on surgical practice. J Br Soc Echocardiogr.

[2] Couture P, Denault AY, McKenty S, Boudreault D, Plante F, Perron R (2000). Impact of routine use of intraoperative transesophageal echocardiography during cardiac surgery. Can J Anesth.

[3] Mishra M, Chauhan R, Sharma KK, Dhar A, Bhise M, Dhole S (1998). Real-time intraoperative transesophageal echocardiography--how useful?.Experience of 5,016 cases. J Cardiothorac Vasc Anesth.

